# Fornix White Matter is Correlated with Resting-State Functional Connectivity of the Thalamus and Hippocampus in Healthy Aging but Not in Mild Cognitive Impairment – A Preliminary Study

**DOI:** 10.3389/fnagi.2015.00010

**Published:** 2015-02-05

**Authors:** Elizabeth G. Kehoe, Dervla Farrell, Claudia Metzler-Baddeley, Brian A. Lawlor, Rose Anne Kenny, Declan Lyons, Jonathan P. McNulty, Paul G. Mullins, Damien Coyle, Arun L. Bokde

**Affiliations:** ^1^Trinity College Institute of Neuroscience and Cognitive Systems Group, Discipline of Psychiatry, School of Medicine, Trinity College Dublin, Dublin, Ireland; ^2^Cardiff University Brain Research Imaging Centre (CUBRIC), Neuroscience and Mental Health Research Institute (NMHRI), School of Psychology, Cardiff University, Cardiff, UK; ^3^Department of Psychiatry, Jonathan Swift Clinic, St. James Hospital, Trinity College Institute of Neuroscience, Trinity College Dublin, Dublin, Ireland; ^4^Mercer’s Institute for Successful Ageing, St. James Hospital, Trinity College Institute of Neuroscience, Trinity College Dublin, Dublin, Ireland; ^5^St. Patrick’s Hospital, Dublin, Ireland; ^6^School of Medicine and Medical Science, University College Dublin, Dublin, Ireland; ^7^School of Psychology, Bangor University, Bangor, UK; ^8^Intelligent Systems Research Centre, University of Ulster, Derry, UK

**Keywords:** diffusion MRI, tractography, functional connectivity, fornix, mild cognitive impairment (MCI), hippocampus, thalamus

## Abstract

In this study, we wished to examine the relationship between the structural connectivity of the fornix, a white matter (WM) tract in the limbic system, which is affected in amnestic mild cognitive impairment (aMCI) and Alzheimer’s disease, and the resting-state functional connectivity (FC) of two key related subcortical structures, the thalamus, and hippocampus. Twenty-two older healthy controls (HC) and 18 older adults with aMCI underwent multi-modal MRI scanning. The fornix was reconstructed using constrained-spherical deconvolution-based tractography. The FC between the thalamus and hippocampus was calculated using a region-of-interest approach from which the mean time series were exacted and correlated. Diffusion tensor imaging measures of the WM microstructure of the fornix were correlated against the Fisher *Z* correlation values from the FC analysis. There was no difference between the groups in the fornix WM measures, nor in the resting-state FC of the thalamus and hippocampus. We did however find that the relationship between functional and structural connectivity differed significantly between the groups. In the HCs, there was a significant positive association between linear diffusion (CL) in the fornix and the FC of the thalamus and hippocampus, however, there was no relationship between these measures in the aMCI group. These preliminary findings suggest that in aMCI, the relationship between the functional and structural connectivity of regions of the limbic system may be significantly altered compared to healthy ageing. The combined use of diffusion weighted imaging and functional MRI may advance our understanding of neural network changes in aMCI, and elucidate subtle changes in the relationship between structural and functional brain networks.

## Introduction

Alzheimer’s disease (AD) is the most common cause of neurodegenerative dementia and is often preceded by a stage known as amnestic mild cognitive impairment (aMCI) (Petersen et al., 1999; Petersen, 2004), which confers an increased risk of developing AD (Stephan et al., 2012). With the prevalence of AD predicted to rise substantially over the coming years (Barnes and Yaffe, 2011), there has been increasing interest in using neuroimaging to understand early brain changes associated with preclinical groups such as aMCI (Jack et al., 2010; Sperling et al., 2011), which may represent the earliest stages of the disease. Early neuroimaging studies of aMCI and AD predominantly focused on localized brain changes such as hippocampal atrophy (Hua et al., 2010; Jack et al., 2010), however there is now a large body of evidence suggesting that the brain’s inherent structural and functional connectivity (FC) is disrupted in aMCI and AD (Seeley et al., 2009; Teipel et al., 2014; Vidal-Pineiro et al., 2014).

Functional connectivity studies have found reduced connectivity in resting-state networks in aMCI and AD, including the default mode network (DMN) (Greicius et al., 2004; Beason-Held, 2011; Brier et al., 2012; Jacobs et al., 2013; Sheline and Raichle, 2013), and have found that altered FC is also linked to memory dysfunction in these populations (Wu et al., 2013; Dunn et al., 2014). Studies using diffusion weighted imaging (DWI) and tractography to measure white matter (WM) connectivity in aMCI and AD have found significant alterations in tracts essential for memory function such as the fornix, uncinate fasciculus, and cingulum (Naggara et al., 2006; Muller et al., 2007; Stahl et al., 2007; Catheline et al., 2010; Fellgiebel and Yakushev, 2011; Metzler-Baddeley et al., 2012a,b).

The fornix is the main efferent pathway of the hippocampus, connecting it with regions including the mammillary bodies of the hypothalamus and the thalamus (Schmahmann and Pandya, 2006; Aggleton et al., 2010). A number of studies have found changes in the fornix in aMCI and AD (Mielke et al., 2009; Zhuang et al., 2010; Bozoki et al., 2012; Lee et al., 2012), such as lower fractional anisotropy (FA – thought to reflect fiber tract density and myelination). Furthermore, in a prospective study in healthy older adults, lower FA in the fornix at baseline predicted conversion to aMCI after 2 years (Zhuang et al., 2012a).

Although memory dysfunction in aMCI has been linked with both hippocampal atrophy (Convit et al., 1997; Grundman et al., 2003; Stoub et al., 2006) and WM degeneration in the fornix (Zhuang et al., 2012b), however, the role of the thalamus in aMCI is less well-defined, even though this structure is closely associated with the fornix and hippocampus. The thalamus plays a major role in generating the many rhythms in electroencephalography (EEG), which change substantively during neurodegeneration (Cantero et al., 2009); yet little is known about the role of the thalamus in neurodegeneration, and whether or not thalamus atrophy is a primary or secondary phenomenon to hippocampal or cortical atrophy in AD.

Several volumetric studies have found that the thalamus does undergo neurodegeneration in aMCI and AD (Chetelat et al., 2005; Shiino et al., 2006; de Jong et al., 2008; Cherubini et al., 2010; Roh et al., 2011; Zhang et al., 2013), and in a study that combined shape analysis of the thalamus and diffusion tensor imaging (DTI) (Zarei et al., 2010), thalamic regions most highly connected to the hippocampus showed the most severe atrophy in aMCI. Changes in the FC of the thalamus have been reported in aMCI (Zhou et al., 2013) albeit less frequently than changes in hippocampal FC, and in one study of healthy elders (Ystad et al., 2010), a negative correlation was found between thalamic FC and verbal free recall. This suggested that higher performers displayed more de-synchronization of thalamic signals, and that the FC of the thalamus may be linked to memory function. Yoon et al. (2012) found a positive correlation between perfusion in the left thalamus and performance on the Rey complex figure test in MCI, suggesting a role for the thalamus in cognitive decline.

In the current study, we used DWI and resting-state functional MRI (fMRI) to examine the connectivity of the fornix, thalamus, and hippocampus in aMCI, putatively viewed as a set of connected brain structures, which together form part of a limbic episodic memory network that malfunctions if one or more components are impaired. In particular, we wished to examine whether there was a correspondence between the structural and FC measures, and whether this was altered in aMCI. This is the first study to our knowledge to examine the link between structural and FC of the thalamus and hippocampus in aMCI. Previous studies have suggested that functional and structural connectivity are closely related, particularly in healthy participants (Damoiseaux and Greicius, 2009; Greicius et al., 2009), however the link between the two is not well-defined (Sporns, 2014), especially in the context of ageing and aMCI. Disruption of anatomical connections may influence the organization of FC, and combining MRI modalities should provide greater insight into the neural network connectivity changes in aMCI.

We performed constrained-spherical deconvolution (CSD)-based fiber tractography of the fornix in a cohort of older adults with aMCI and healthy age-matched controls, and examined the resting-state FC of the thalamus and hippocampus. Taken together, these MRI measures should provide a fuller picture of the changes that occur in the fornix in aMCI, as well as about the relationships between microstructural WM changes and alterations in FC. We predicted that the aMCIs would show decreased FA (thought to reflect fiber tract density and myelination; Mori and Zhang, 2006), and increased mean diffusivity values (MD – which tends to be low in highly intact, organized tracts and which usually increases in disease states and neurodegeneration; Mori and Zhang, 2006) in the fornix relative to controls, in line with previous studies (Mielke et al., 2009; Zhuang et al., 2010; Bozoki et al., 2012; Lee et al., 2012). We also predicted that there would be reduced resting-state FC of the thalamus and hippocampus, similar to previous findings (Zhou et al., 2008, 2013), which may be related to WM microstructural alterations in the fornix.

## Materials and Methods

### Participants

Twenty-two older healthy control (HC) participants and 19 older participants with aMCI took part in the study. The tractography analysis was unsuccessful for one aMCI participant, in whom the tracts were almost completely absent. An examination of their T1-weighted anatomical scan revealed quite advanced levels of atrophy and gross enlargement of the lateral ventricles. Therefore, 18 aMCI were included in the final sample.

The HCs were community-dwelling older adults recruited from the greater Dublin area *via* newspaper advertisements. They underwent a health screening questionnaire and a neuropsychological assessment, the Consortium to Establish a Registry for Alzheimer’s Disease (CERAD, Morris et al., 1988), in order to rule out possible cognitive impairment before inclusion in the study. The CERAD battery has been shown to be sensitive to the presence of age-related cognitive decline (Welsh et al., 1991, 1992). All of the older participants included in the study scored no more than 1.5 SD below the standardized mean scores for subjects of a similar age and education level on any of the sub-tests.

The aMCI participants were recruited from memory clinics in St. James Hospital and St. Patrick’s Hospital in Dublin, and were diagnosed by a clinician according to the Peterson criteria (Petersen et al., 1999) – i.e., abnormal memory scores for age and education level with no dementia. Four were single amnestic MCI (aMCI), and 14 were multi-domain aMCI (Petersen, 2004). Neuropsychological measures were administered or supervised by an experienced neuropsychologist and included the mini-mental state examination (MMSE; Folstein et al., 1975) and Cambridge cognitive examination (CAMCOG; Huppert et al., 1995).

All of the participants were right-handed with no history of head trauma, neurological disease, stroke, transient ischemic attack, heart attack, or psychiatric illness. They completed the Geriatric Depression Scale (GDS; Yesavage, 1988), the Eysenck Personality Questionnaire Revised Edition Short Scale (EPQ-R; Eysenck and Eysenck, 1991), and a Cognitive Reserve Questionnaire (Rami et al., 2011) before the MRI scan. The groups did not differ in terms of age, gender, education level, or levels of cognitive reserve as assessed by the self-report Cognitive Reserve Questionnaire. The aMCI group had lower MMSE scores, higher GDS scores, and scored lower on the EPQ measure of extraversion than the HC group. See Table 1 for a summary of the participant demographics.

**Table 1:** **Demographic details of the participants**.

	HC (*n* = 22)	aMCI (*n* = 18)	p* (*df* = 38)
Gender	12 M, 10 F	9 M, 9 F	1.00
Age	68.86 ± 6.47	68.83 ± 7.71	0.99
Education	14.36 ± 3.17	14.50 ± 3.00	0.89
MMSE	28.82 ± 0.96	27.22 ± 2.10	**0.003**
GDS	0.77 ± 1.11	2.67 ± 2.30	**0.002**
EPQ E	8.27 ± 2.64	5.33 ± 3.36	**0.004**
EPQ N	2.14 ± 0.96	3.89 ± 3.46	0.52
CR	17.82 ± 3.02	16.89 ± 4.92	0.47

*MMSE, mini-mental state exam; GDS, geriatric depression scale; EPQ E, Eysenck personality questionnaire extraversion scale; EPQ N, Eysenck personality questionnaire neuroticism scale; CR, cognitive reserve scale. Standard deviations are indicated in parentheses*.

*Results of independent samples *t*-tests, except for gender which was compared with a Fischer’s exact test. Statistically significant differences are indicated in bold font.

The study had full ethical approval from the St. James Hospital and the Adelaide and Meath Hospital, incorporating the National Children’s Hospital Research Ethics Committee. All participants gave written informed consent before taking part in the study.

### MRI data acquisition

Whole-brain high angular resolution diffusion imaging (HARDI) data were acquired on a 3.0 Tesla Philips Intera MR system (Best, The Netherlands) equipped with an eight channel head coil. A parallel sensitivity encoding (SENSE) approach (Pruessmann et al., 1999) with a reduction factor of two was used during the DWI acquisition. Single-shot spin echo-planar imaging was used to acquire the DWI data with following parameters: Echo time (TE) 79 ms, repetition time (TR) 20,000 ms, field of view (FOV) 248 mm, matrix 112 × 112, isotropic voxel of 2.3 mm × 2.3 mm × 2.3 mm, and 65 slices with 2.3 mm thickness with no gap between the slices. Diffusion gradients were applied in 61 isotropically distributed orientations with *b* = 3000 s/mm^2^, and four images with *b* = 0 s/mm^2^ were also acquired. The total scan time was 17 min.

A high-resolution 3D T1-weighted anatomical image was acquired for each participant with the following parameters: TE = 3.9 ms, TR = 8.5 ms, FOV = 230 mm, slice thickness = 0.9 mm, voxel size = 0.9 mm × 0.9 mm × 0.9 mm. These images were used for the correction of EPI-induced geometrical distortions in the DWI data.

Resting-state fMRI data were also acquired during the scanning session. The scan lasted for 7 min during which time the participants were asked to keep their eyes open and fixate on a cross hairs in the center of a screen behind the MR scanner, visible *via* a mirror. The blood oxygenation dependent (BOLD) signal changes were measured using a T2*-weighted echo-planar imaging sequence with TE = 30 ms and TR = 2000 ms. Each volume of data covered the entire brain with 39 slices, and the slices were acquired in interleaved sequence from inferior to superior direction. Two hundred ten volumes of data were acquired, with voxel dimensions of 3.5 mm × 3.5 mm × 3.85 mm and a 0.35 mm gap between the slices.

### DWI analysis

The DWI data were analyzed using ExploreDTI v4.8.3 (Leemans et al., 2009)^1^. The images were corrected for distortion due to head motion, eddy currents and for EPI-induced geometrical distortions by co-registration and resampling to the high-resolution T1-weighted anatomical images. This was implemented in ExploreDTI according to the method described by Irfanoglu et al. (2012) and the encoding vectors were reoriented appropriately (Leemans and Jones, 2009). Since ageing and neurodegeneration are related to brain atrophy and the fornix is extremely susceptible to contamination from cerebrospinal fluid (CSF) and atrophy-based partial volume artefacts, the free water elimination approach (Pasternak et al., 2009) was applied to correct for partial volume effects prior to fitting the tensor model to the data in each voxel. The free water elimination method has been used successfully in several previous tractography studies of ageing and aMCI, particularly in relation to the fornix (Metzler-Baddeley et al., 2012a,b; Fletcher et al., 2014).

#### Tractography of the fornix

For the analysis of the fornix a hybrid analysis approach was used, whereby reconstruction of the tracts was completed using CSD-based tractography (Jeurissen et al., 2011) and DTI-based WM indices were extracted for statistical analyses. CSD rather than DTI-based tractography was chosen as it can account for complex WM orientation such as crossing fibers (Tournier et al., 2008, 2011), and this approach has recently been successful at detecting changes in tracts with complex WM architecture in MCI and AD (Metzler-Baddeley et al., 2012b; Reijmer et al., 2012). In the case of the fornix for instance, DTI-based tractography cannot resolve complex fiber architecture in regions where the anterior columns of the fornix cross with fibers of the anterior commissure (Metzler-Baddeley et al., 2012a). Several previous studies have successfully used deterministic tractography based on the CSD method to segment the fornix (Metzler-Baddeley et al., 2011, 2013).

After the pre-processing steps whole-brain tractography was performed using every voxel as a seed point. The principle diffusion orientation at each point was estimated by the CSD tractography algorithm, which propagated in 0.5 mm steps along this direction. At each new location the fiber orientation(s) was estimated before the tracking moved a further 0.5 mm along the direction that subtended the smallest angle to the current trajectory. A trajectory was followed through the data until the scaled height of the fiber orientation density function peak dropped below 0.1, or the direction of the pathway changed through an angle of more than 60°.

**Figure 1:**
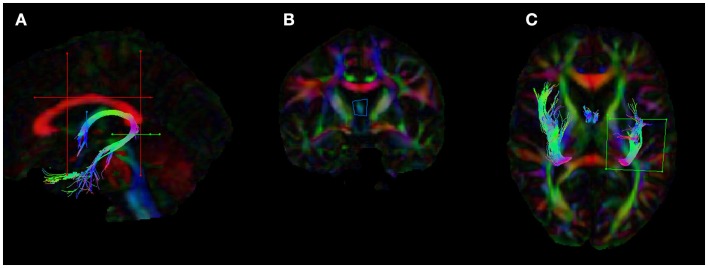
**Placement of the regions of interests (ROIs) for the dissection of the fornix in ExploreDTI**. **(A)** The five ROIs used to segment the fornix **(B)** coronal view of the seed ROI **(C)** ROI used to split the tracts and isolate the left fornix. The blue ROI is a SEED/OR gate, the green ROI is an AND gate, whilst the red ROIs are NOT gates. These ROIs were drawn for one subject and once deemed to be accurate and robust they were applied to all other subjects using an “atlas-based” tract segmentation method. The atlas in this case was the FA image of this subject.

Following whole-brain tractography, the fornix was extracted by drawing several regions of interest (ROIs) defined according to previously published methods (Metzler-Baddeley et al., 2011, 2013). The ROIs were drawn manually for one subject and then applied to all of the other subjects using an “atlas-based” tractography (ABT) approach, by spatial transformation of the ROIs to the other subjects’ native space. This ensured consistency in the placement of the ROIs. See Figure 1 for an example of the ROIs in the template subject, which was a HC participant. For several subjects this ABT approach was unsuccessful, most often due to inter-subject anatomical variability, such as neurodegeneration and encroachment of the lateral ventricles in some of the aMCI participants. In these cases, the ROIs for the tractography were drawn manually, with some adjustment and/or extra ROIs typically needed.

#### Statistical analysis of the tractography data

From the reconstructions of the fornix WM microstructural indices were extracted for statistical analyses in IBM SPSS Statistics for Windows, Version 22.0 (Armonk, NY: IBM Corp). These included free water corrected FA, corrected MD and the corrected Westin measures of linear diffusion coefficient (CL), and planar diffusion coefficient (CP) (Westin et al., 2002). The Westin measures describe the geometrical shape of the diffusion tensor, with a high value of CL implying only one dominant fiber orientation within a voxel (Vos et al., 2012), and a high value of CP indicating the presence of crossing fiber configurations (Vos et al., 2012).

The diffusion tensor metrics in the left and right fornix were compared within the two groups using a series of paired *t*-tests and no statistically significant differences were found. The values in the left and right fornix were therefore averaged to give a single fornix measure for each subject. This also reduced the number of multiple comparisons in the statistical tests, reducing the possibility of Type I errors. The diffusion metrics were compared between the groups using four paired *t*-tests, which were corrected for multiple comparisons using a Bonferroni-corrected *p*-level of *p* < 0.0125.

### Resting-state functional connectivity of the thalamus and hippocampus

The fMRI data were processed using the DPARSF V2.3 toolbox (Data Processing Assistant for Resting-State fMRI, Yan and Zang, 2010)^2^, which utilizes SPM8^3^ for the pre-processing steps and the REST V1.8 toolbox (Song et al., 2011) for the resting-state analysis. Data pre-processing involved slice timing correction, realignment to correct for head motion, normalization to MNI152 space by T1-image unified segmentation, smoothing with a 4 mm full-width-at-half-maximum Gaussian kernel, and detrending and filtering (0.01–0.08 Hz). The normalized voxel size was 3 mm × 3 mm × 3 mm. Several nuisance covariates were regressed out, including six head motion parameters and signals from the WM and CSF.

**Figure 2:**
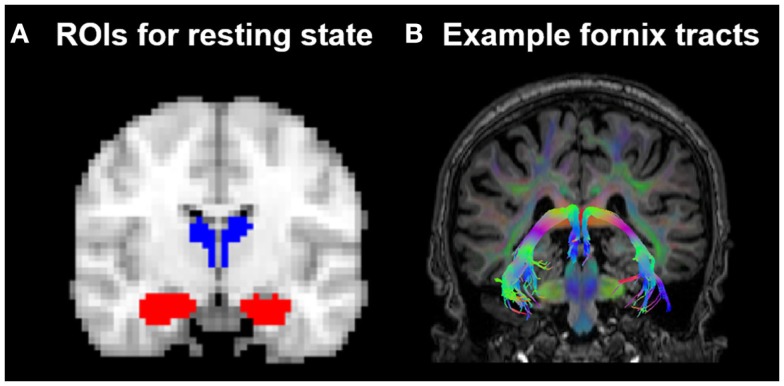
**(A)** Position of the resting-state regions of interest (ROIs) in relation to **(B)** the fornix in one HC subject. ROIs for the resting-state analysis were placed in the hippocampus (indicated in red) and the thalamic region probabilistically connected to the temporal lobe (indicated in blue), as defined by the Oxford thalamic connectivity atlas (Behrens et al., 2003a,b).

The resting-state FC of the thalamus and hippocampus was measured using a ROI FC approach. The ROIs were created using probabilistic atlases included in FSL^4^ and were defined in the MNI152 template space. The left and right hippocampus ROIs were based on the Harvard-Oxford subcortical atlas structures. For the thalamus ROIs, the Oxford thalamic connectivity atlas (Behrens et al., 2003a,b), a probabilistic atlas of seven sub-thalamic regions, segmented according to their WM connectivity to cortical areas, was used to delineate the temporal region of the thalamus. The temporal region was chosen because, anatomically, this region is most likely to be structurally connected to the cortex nearest the hippocampus, and so the temporal thalamic-hippocampus resting-state measure was devised to mirror as closely as possible the regions structurally connected by the fornix. See Figure 2. The four subcortical ROIs were thresholded at a minimum probability level of 20%, binarized, and re-sampled to 3 mm × 3 mm × 3 mm.

The average fMRI time series from the four ROIs were extracted and correlated to determine the FC of the regions at rest. Fisher-*Z* score transformations of these values were exported to IBM SPSS v.22 for second-level statistical analysis. The FC Fischer *Z*-scores between the left thalamus and left hippocampus, and between the right thalamus and right hippocampus, were compared within groups, and there was found to be no statistically significant difference. An average thalamus–hippocampus FC measure was therefore calculated for each subject, which was correlated against the fornix WM measures. For each group, four correlations were run and a Bonferroni-corrected *p*-value of 0.0125 was applied.

Several of the correlation coefficients were compared between the groups. To do this, the Pearson *r* values were first converted to Fisher *Z* scores. The *Z* statistic was then calculated using:

$Z=\left(\mathrm{Fisher}\;Z1-\mathrm{Fisher}\;Z2\right)∕\sqrt{\left(\mathrm{variance}\right)},$ where variance is equal to: $\frac{1}{N1}+\frac{1}{N2}$.

## Results

### Comparison of fornix white matter microstructure in HC and aMCI groups

**Figure 3:**
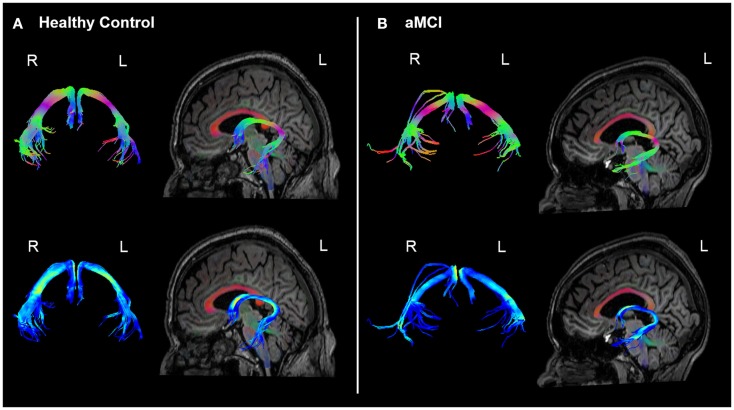
**Example fornix tracts in a (A) healthy control and (B) amnestic aMCI subject**. The top panel in each case shows the tracts color-encoded with the first eigenvector (FE); the bottom panel shows the same tracts color-encoded with FA values. For each subject, the left and right fornix is shown in isolation on the left, and on the right the fornix can be seen overlaid on the subject’s T1-weighted structural image, with the FE shown in semi-transparent color (note the corpus callosum in red for example). The DWI data were co-registered to the structural images during the processing to correct for EPI-induced geometric distortions, however as illustrated by these figures this also facilitates the inspection of the white tracts in reference to the high-resolution anatomical image of the brain.

The analyses of WM microstructural indices in the fornix revealed no statistically significant differences between the HC and aMCI groups. There was a trend towards higher CL and lower CP in the HCs versus aMCIs, however these results did not meet the Bonferroni-corrected threshold of *p* < 0.0125. See Table 2 for a summary of these results and Figure 3 for examples of the fornix tracts in one HC and one aMCI participant.

**Table 2:** **Mean DWI measures for fornix in the HC and aMCI groups**.

Fornix	HC	aMCI	*t*-statistic	*p*-value
FA	0.25 ± 0.02	0.24 ± 0.03	1.22	0.23
MD	0.0009 ± 0.00006	0.0009 ± 0.00007	−0.88	0.38
CL	0.29 ± 0.02	0.26 ± 0.04	2.21	0.033
CP	0.0698 ± 0.012	0.0863 ± 0.032	−2.21	0.034

*FA, fractional anisotropy; MD, mean diffusivity; CL, linear diffusion coefficient; CP, planar diffusion coefficient*.

### Resting-state functional connectivity of the thalamus and hippocampus and relation to fornix WM microstructure

The average FC of the temporal thalamic regions and the hippocampus indicated a high degree of resting-state FC in both the HCs (Fisher’s *Z* = 0.48 ± 0.23) and aMCIs (Fisher’s *Z* = 0.50 ± 0.21). There was no statistically significant difference in FC between the groups [*t* (38) = −0.32, *p* = 0.75].

**Figure 4:**
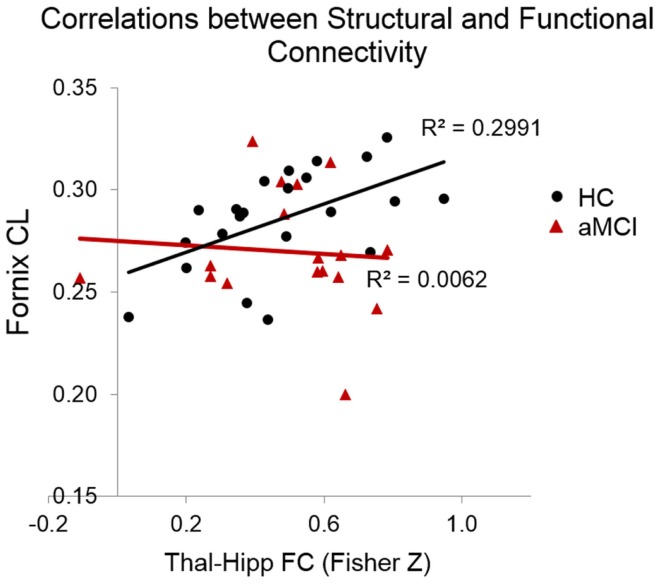
**Scatterplots indicating the correlations between functional and structural connectivity measures**. In the HCs but not the MCIs there was a significant positive relationship between CL in the fornix and the resting-state connectivity of the thalamus and hippocampus.

The correlational analysis of the functional and structural connectivity measures revealed a significant positive association in the HC group between the linear diffusion coefficient in the fornix and the FC of the thalamus–hippocampus (*r* = 0.55, *p* = 0.008), which was absent in the aMCI group (*r* = −0.08, *p* = 0.81). These correlation coefficients differed significantly from one another (*Z* = 5.17, *p* < 0.0001). Scatterplots of these correlational results are shown in Figure 4. One of the datasets had a CL value of <0.2, which upon examination was found to be a statistical outlier (*p* < 0.05). This dataset was removed from the correlational analyses, thus in Figure 4 there are 22 HCs and 17 aMCI datasets plotted.

## Discussion

In this study, we examined the structural integrity of the fornix, an important limbic WM tract, and the resting-state FC of two associated subcortical structures, the thalamus, and hippocampus. This is the first study to our knowledge to examine the relationship between the structural and FC of the thalamus and hippocampus, and whether this is altered in aMCI, a condition which is known to affect fornix WM and resting-state connectivity of intrinsic brain networks.

### Relationship between functional and structural connectivity

Although, contrary to our predictions, we found no evidence of reduced FC between the thalamus and hippocampus in aMCI, we did find that the correspondence between the FC and fornix WM was altered in this group. There was a significant positive relationship in the HCs between CL in the fornix and the FC of the thalamus–hippocampus, however this relationship was absent in the aMCIs.

Elucidating the relationship between structural and FC is not trivial, and is an area of growing interest within the neuroimaging community (van den Heuvel and Sporns, 2013; Sporns, 2014). Previous combined fMRI–DTI studies have found that resting-state FC does in general reflect the brain’s structurally connected WM networks (Damoiseaux and Greicius, 2009; Greicius et al., 2009), and functional and structural connectivity measures have been found to correlate, particularly in healthy individuals (Skudlarski et al., 2008; van den Heuvel et al., 2008).

Several studies have indicated however, that the relationship between functional and structural connectivity can be altered in disease states. For example, in a study of major depressive disorder (de Kwaasteniet et al., 2013), patients had a negative correlation between FA in the uncinate fasciculus – another of the limbic WM tracts – and the FC between the subgenual anterior cingulate cortex and the hippocampus, which was not mirrored in the HC. Furthermore, in a study of schizophrenia (Skudlarski et al., 2010), patients compared to controls were found to show not only overall reduced global WM connectivity as derived from DTI, but also a lower coherence between WM connectivity and FC. The current study supports the idea that the correspondence between functional and structural connectivity may be altered in neuropathological states. This is an area of research which certainly warrants further investigation in future studies.

Functional connections in the brain also exist between regions, which are not directly structurally connected, likely mediated by indirect structural connections (Honey et al., 2009), however in the current study we focused our analysis on the connectivity of two subcortical structures, which are known to be directly linked *via* a WM tract. We found evidence that there is a close correspondence between functional and structural connectivity in these structures in healthy ageing, which appears to be disrupted in aMCI.

### Fornix results

A number of previous studies have found that the fornix is affected in aMCI, with changes such as lower FA and increased RD reported (Lee et al., 2012; Metzler-Baddeley et al., 2012a; Oishi et al., 2012; Zhuang et al., 2013). We had predicted that the aMCI group would show similar changes in fornix WM indices consistent with compromised WM, however we found no evidence of this in the current study, with no differences in fornix WM microstructural indices between the groups.

The lack of difference in the fornix WM microstructural indices between the HCs and aMCIs may be due to sparing of the fornix in this aMCI cohort, however several other factors may also have contributed. This study was limited by a relatively small sample size, which likely reduced the power to detect group differences. Furthermore, the aMCI cohort included both single and multi-domain aMCI. This increased heterogeneity of the sample may also have decreased the likelihood of detecting differences between the groups. Multi-domain aMCI also confers an increased risk of conversion to vascular dementia (VaD) (Petersen, 2004; Rasquin et al., 2005; Libon et al., 2010) and not just AD, which has typically not been found to involve the same level of disruption to the limbic system as AD (Burton et al., 2009). However some previous studies of aMCI also failed to detect changes in FA (Nowrangi et al., 2013; Rowley et al., 2013), suggesting that FA may not always be a strong indicator of WM connectivity changes in this cohort.

### Linear and planar diffusion coefficients

The WM indices of linear and planar diffusion coefficients (CL and CP) did show a trend towards being significantly different between the groups (*p* < 0.035 in each case, which did not survive Bonferroni correction), with higher CL and lower CP in the HCs. High CL values are thought to reflect the presence of one dominant fiber orientation within a voxel (Vos et al., 2012), while high CP values are thought to indicate the presence of crossing fiber configurations (Vos et al., 2012). The fornix is generally considered to be a largely single-fiber orientation tract, however differences in CL and CP may also reflect WM damage. For example, in a recent study, which investigated WM changes following a season of playing varsity football (Davenport et al., 2014), there was a strong association between the number of head impacts and reduced FA, as well as reduced CL and increased CP. In fact, the strongest statistical relationship was with changes in CL, with the authors suggested may reflect WM disconnection due to the focal disruption of axons caused by the traumatic head impacts. Changes in CL and CP may also reflect WM changes in aMCI, such as WM degeneration and disruption to axonal organization, however further investigation of how these indices change in ageing and aMCI is needed.

### Strengths and limitations of the current study

The present study extends previous studies by examining how fornix WM microstructural is related to the resting-state FC of two closely related structures, the thalamus and hippocampus. It is becoming common for neuroimaging researchers to collect multi-modal MRI data, therefore it is important for more studies to integrate findings across these modalities. This is the first study to our knowledge to combine FC and diffusion tractography to examine the fornix in ageing and aMCI, and the first to find differences in the relationship between the functional and structural connectivity of the thalamus and hippocampus related to aMCI.

The DWI methods employed in this study were very robust, as CSD-based tractography methods are preferable to DTI-based approaches, particular for brain regions with complex WM architecture. Correcting for free water is also an important pre-processing step since it is well established that atrophy-related CSF partial volume can bias DTI-based indices (Metzler-Baddeley et al., 2012a; Vos et al., 2012; Baron and Beaulieu, 2014; Maier-Hein et al., 2014). A recent study has highlighted how signals from CSF can contribute to differences in WM microstructural measures in MCI (Berlot et al., 2014), highlighting the importance of controlling for this confound.

A possible methodological limitation of our study is that we did not examine possible non-linear relationships between WM measures and FC. Variability in these measures may also be better captured by using more advanced statistical approaches, such as joint ICA (Calhoun et al., 2009). This is something which could be explored in future studies.

## Conclusion

In the current study, we used multi-modal MRI to examine the relationship between the functional and structural connectivity of two important subcortical structures, the thalamus and hippocampus, in healthy ageing and aMCI. We did not replicate previous findings of changes in DTI metrics in aMCI, which may indicate that the study was underpowered to properly test these differences. Thus the results should be taken as preliminary findings, which we believe warrant further investigation in a larger cohort. However, although there were no group differences in the WM measures of the fornix or in the FC of the thalamus–hippocampus at rest, there was a strong correspondence between the structural and FC measures in the HCs, which was absent in the aMCI group. The results suggest a disruption to the relationship between functional and structural connectivity in aMCI, which may be representative of early neuropathological connectivity changes in the limbic system. Both DWI and FC have offered new insights into brain network changes in aMCI. However, the complementary strengths of both of these methods combined may advance our understanding of neural network disconnection in this condition, and may increase the potential biomarker capabilities of MRI by elucidating subtle changes in the relationship between structural and functional brain networks.

## Conflict of Interest Statement

The authors declare that the research was conducted in the absence of any commercial or financial relationships that could be construed as a potential conflict of interest.

## Footnotes

^1^http://www.exploredti.com/

^2^http://rfmri.org/DPARSF

^3^http://www.fil.ion.ucl.ac.uk/spm

^4^http://fsl.fmrib.ox.ac.uk/fsl/fsl4.0/fslview/atlas-descriptions.html
